# Biomolecule-Based Coacervation: Mechanisms, Applications, and Future Perspectives in Biomedical and Biotechnological Fields

**DOI:** 10.3390/biom15060861

**Published:** 2025-06-13

**Authors:** Dong Hyun Kim, Mi-Ran Ki, Da Yeon Chung, Seung Pil Pack

**Affiliations:** 1 Department of Biotechnology and Bioinformatics, Korea University, Sejong 30019, Republic of Korea; jklehdgus@korea.ac.kr (D.H.K.); anthic83@korea.ac.kr (D.Y.C.); 2 Institute of Industrial Technology, Korea University, Sejong 30019, Republic of Korea; allheart@korea.ac.kr

**Keywords:** liquid–liquid phase separations, coacervates, membraneless organelles, complex coacervation, simple coacervation

## Abstract

Coacervate is a form of liquid–liquid phase separation (LLPS) in which a solution containing one or more charged components spontaneously separates into two immiscible liquid phases. Due to their ability to mimic membraneless cellular environments and their high biocompatibility, coacervates have found broad applications across various fields of life sciences. This review provides a comprehensive overview of recent advances in biomolecule-based coacervation for biotechnological and biomedical applications. Encapsulation via biomolecule-based coacervation enables high encapsulation efficiency, enhanced stability, and the sustained release of cargos. In the field of tissue engineering, coacervates not only support cell adhesion and proliferation but also serve as printable bioinks with tunable rheological properties for 3D bioprinting. Moreover, biomolecule-based coacervates have been utilized to mimic membraneless organelles, serving as experimental models to understand the origin of life or investigate the mechanisms of biochemical compartmentalization. This review discusses the mechanisms of coacervation induced by various types of biomolecules, evaluates their respective advantages and limitations in applied contexts, and outlines future research directions. Given their modularity and biocompatibility, biomolecule-based coacervates are expected to play a pivotal role in next-generation therapeutic development and the construction of controlled tissue microenvironments, especially when integrated with emerging technologies.

## 1. Introduction

Coacervation is liquid–liquid phase separation (LLPS) that occurs in a solution containing one or more polymeric colloids that spontaneously separate into two immiscible liquid phases [1]. In this process, the phase with a higher concentration of colloidal components is referred to as the coacervate phase, which exists in equilibrium with a lower-density liquid phase [2]. The coacervate phase typically appears as amorphous liquid droplets and, over time, these droplets can fuse together, forming a more stable structure that can be obtained in large quantities [1,3]. Due to these physicochemical properties, coacervation has found various applications in the biological and biomedical fields, where it serves as a crucial mechanism for molecular organization and regulation under physiological conditions [4,5,6].

In biological systems, the organization and compartmentalization of biomolecules play an essential role in maintaining cellular function [7]. Traditionally, intracellular compartmentalization has been attributed mainly to the membrane organelles [8,9]. However, recent studies have highlighted the significance of LLPS-driven compartmentalization as an alternative mechanism for organizing cellular components [3]. LLPS enables the selective enrichment and segregation of biomolecules, leading to the formation of coacervate phases [10]. As a result, biomolecule-based coacervation has attracted increasing attention in life sciences and bioengineering, particularly for its role in various biological processes.

Biomolecule-based coacervation is driven by interactions among biomolecules such as proteins, nucleic acids, and polysaccharides. These interactions involve electrostatic attractions, hydrogen bonding, and hydrophobic forces [10,11,12,13]. Compared to synthetic polymer-driven systems, biomolecule-based coacervates offer superior biocompatibility, greater responsiveness to physiological stimuli, and intrinsic functional significance for biological systems [8,14]. For example, protein-based coacervates can create microenvironments that regulate enzyme activity, while nucleic acid-based coacervates play crucial roles in gene expression and RNA metabolism [15,16,17].

Although synthetic polymer-based coacervation systems have been widely explored, they often exhibit low biocompatibility and pose challenges related to polymer residue and purification [1,11]. In contrast, biomolecule-based coacervates provide a more biomimetic and biologically relevant platform, exhibiting features like stimuli-responsive behavior and enhanced integration with cellular systems [18,19,20].

**Figure 1:**
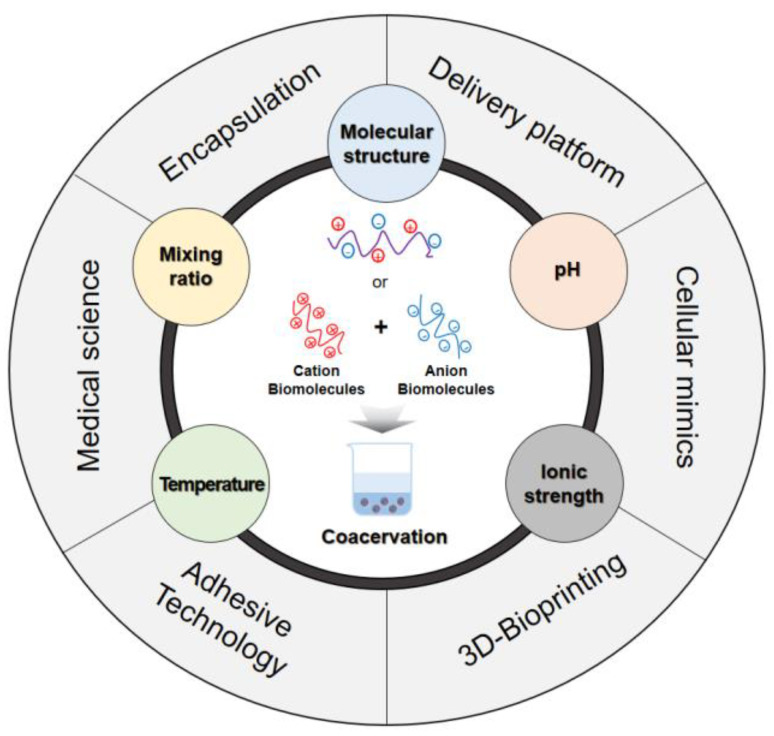
Illustration of factors and applications of coacervation based on cation biomolecules and anion biomolecules.

Given these advantages, biomolecule-based coacervation is emerging as a key research focus in fields such as synthetic biology, biotechnology, and biomedicine. Despite the growing interest and recent progress in this system, a comprehensive understanding of its biotechnological and biomedical applications remains limited. As shown in Figure 1, this review summarizes the mechanisms, types, and formation factors of coacervation, as well as recent advances in this area, focusing on the biological relevance and application potential of coacervation systems derived from natural biomolecules.

## 2. Mechanism of Coacervation

Various theories have been proposed to understand the complex mechanism of coacervation. Bungenberg de Jong and Kruyt were pioneers in the study of coacervation, first describing the phenomenon in the late 1920s [2]. Their research revealed that polymeric coacervates consist of four non-water species: a polyanion, a polycation, a cation, and an anion. Their work provided essential insights into the interactions between charged polymers and the conditions necessary for coacervation to occur [2]. This early research facilitated the establishment of an empirical basis for explaining the coacervate systems. The first successful theory of complex coacervation was published by Voorn and Overbeek [21]. This model introduced a theoretical framework to describe coacervate systems, highlighting a two-phase coexistence region where phase separation occurs. It described the phenomenon as a spontaneous polymer charge neutralization process driven by electrostatic interactions between the polyelectrolytes used in the experiment. It also explained the conditions for coacervation in terms of polymer charge density, molecular weight, temperature, and dielectric constant [2,21]. Furthermore, this theory described the condition of coacervation based on the Flory–Huggins theory [22,23] for entropy contributions and the Debye–Hückel theory [24] for electrostatic interactions, where *σ* and *r* represent the charge density and polymer molecular weight, respectively [21,25].

$$\sigma^{3}r\geq 0.53$$

Subsequently, Veis and Aranyi refined the Voorn–Overbeek theory by modifying the Flory–Huggins interaction parameter and replacing the electrostatic term with entropy gain to achieve a more precise characterization of coacervation, leading to the development of the following model where *A*^+^ and *B*^−^ represent the polycation and polyanion, respectively [26,27].

$$\begin{array}{c} \left[A^{+}\right]+[B^{-}]\to \;{\left[A^{+}B^{-}\right]}_{agg.}\\ {\left[A^{+}B^{-}\right]}_{agg.}\to \;{\left[A^{+}B^{-}\right]}_{agg.}^{Dilute\;pahse}+{\left[A^{+}+B^{-}\right]}_{Random.C_{c}}^{Conc.\;pahse} \end{array}$$

This theory states that a neutralized aggregate ([*A^+^B^−^*]*_agg._*) is formed through electrostatic interactions, resulting in low configurational entropy. An increase in configurational entropy leads to LLPS that forms randomly mixed concentrated phases and diluted aggregate phases. However, this theory has a limitation in that it is only applicable to polymeric systems with a low charge density.

In 1980, Tainaka advanced the Veis–Aranyi theory by proposing that phase separation in polyelectrolyte systems is primarily driven by attractive forces between aggregates [28]. The Tainaka model expands the applicability beyond low-charge-density systems to include high-charge-density polyelectrolytes, suggesting that coacervation is possible even in systems without specific ionic bonds. Experimental observations indicate that deviating from critical parameters leads to different physical outcomes. When the charge density is excessively high, flocculation occurs instead of coacervation, while insufficient charge density prevents the phase separation necessary for coacervation. However, this theory fails to explain why coacervation is suppressed under low ionic strength conditions and cannot fully describe the complex coacervation processes in multi-polymer systems [2,28].

A variety of contemporary theoretical frameworks have developed to deal with the intricacies of coacervation. These include Random Phase Approximation, Field Theoretic approaches, Counterion Release models, Molecular Simulation techniques, and the Polymer Reference Interaction Site model [29]. These advancements indicate an increasing comprehension of the physical characteristics of polymeric coacervates and the persistent challenges within this field. Although a multitude of theories elucidates various aspects of complex coacervation, no singular theory can thoroughly cover all the characteristics evident in these systems [29]. The variety of approaches signifies continuous investigation and the necessity for an integrated theory capable of forecasting coacervate characteristics across various parameters and scales.

## 3. The Type of Coacervation

Coacervation is classified into simple coacervation and complex coacervation based on its formation process. Simple coacervation involves a single macromolecular species and can be induced by dehydration or desolvation through the addition of salts, alcohols, or other additives, as well as by changes in temperature [18]. This process reduces the interactions between the macromolecule and the solvent while promoting intermolecular interactions among the macromolecules [9,30].

In contrast, complex coacervation is primarily driven by electrostatic interactions between oppositely charged molecules, with charge neutralization being modulated by factors such as ionic strength and pH [31,32]. However, in addition to electrostatic interactions, other intra- and intermolecular forces—such as π–π interactions, cation–π interactions, hydrogen bonding, dipole–dipole interactions, and their combinations—can also influence the coacervation process [33,34].

### 3.1. Simple Coacervation

Simple coacervation, also known as self-coacervation, is a process that occurs when the condition of an aqueous solution is meticulously modulated to specific parameters. These parameters may include the pH level, the ionic strength, or the temperature of the solution. In such instances, the macromolecules within the solution interact intrinsically, resulting in phase separation into a coacervate phase and a dilute phase [18,35].

Cai et al. provided a comprehensive analysis of the molecular mechanisms underlying the simple coacervation of histidine-rich beak protein 1 (HBP-1) and histidine-rich beak protein 2 (HBP-2). Their findings revealed that the process of simple coacervation of HBPs was predominantly driven by hydrophobic interactions, which occurred after charge neutralization through salt screening and pH regulation. An increase in ionic strength was observed to result in enhanced protein–protein interactions, thereby facilitating the process of simple coacervation. This study demonstrated that the sequence modularity of the C-terminus of HBPs played a significant role in their ability to form simple coacervates. This finding suggests that the specific arrangement of amino acids influences their behavior in a solution [36].

Research is also being conducted on simple coacervation utilizing chitosan, a material extensively employed in drug delivery. Jing et al. explored the simple coacervation process of carboxymethyl chitosan (CMCS) [37]. This process was achieved by adjusting the pH of the aqueous solution close to its isoelectric point. The simple coacervation of CMCS occurred across a wide pH range of 3.0 to 6.0, which involved electrostatic interactions, hydrogen bonding, and hydrophobic interactions [37].

Kaushik et al. investigated the simple coacervation of elastin, focusing in particular on the effects of temperature and ionic strength on the coacervation process [38]. The simple coacervation in elastin was explored near its isoelectric point (pI = 4.7). The result of the experiment was that the coacervation temperature was found to decrease from 38 °C to 33 °C as the ionic strength of the solution increased from 0 to 40 mM NaCl. The results indicated that hydrophobic interactions were significant forces in simple coacervation in elastin [38].

### 3.2. Complex Coacervation—Binary and Ternary

Binary coacervation is the most common type of coacervation that is formed through the mixing of two oppositely charged molecules. The stoichiometric properties of two polyelectrolytes, salt, and pH have been demonstrated to exert a significant influence on the processes of their formation, stability, and viscoelastic behavior [1].

Gulão et al. [39] conducted a study on the subject of binary coacervation between polypeptide-leucine and gum arabic (GA), with a view to regulating GA and salt concentration at different pH values ranging from 1.0 to 12.0. The formation of insoluble complex coacervates was most prevalent at a pH of 4.0. The formation of coacervate was indicated by higher turbidity and larger particle sizes in samples containing 0.2% polypeptide-leucine and 0.03% GA without salt. The precipitate was dissociated at a pH of 2.0, which is the pKa of the GA [39].

Ternary coacervation refers to the phase separation process that involves the addition of a third component to the binary coacervation composed of two components. The physical properties and responses of ternary coacervation are largely governed by this third component, which renders its selection critical for maintaining the characteristic features of binary coacervation [18]. The incorporation of a third material has been shown to result in ternary coacervation, thereby providing enhanced protection against environmental factors such as salt concentration and pH. This process enables the formation of the coacervate over a broader compositional range compared to that observed in binary coacervation [18].

Black et al. [40] proposed a method for the stable encapsulation of proteins using complex coacervation driven by electrostatic interactions between charged polypeptides. In this study, PLys was used as the cationic component, while PGlu was employed as the anion counterpart. Bovine serum albumin (BSA) was selected as the third component to induce coacervation. The process of ternary coacervation was initiated by the formation of an intermediate complex between the positively charged PLys and the negatively charged BSA under physiological pH (7.4) through electrostatic interaction. Subsequently, the addition of PGlu to this complex induced ternary coacervation [40].

## 4. Factors Influencing the Coacervation Process

**Figure 2:**
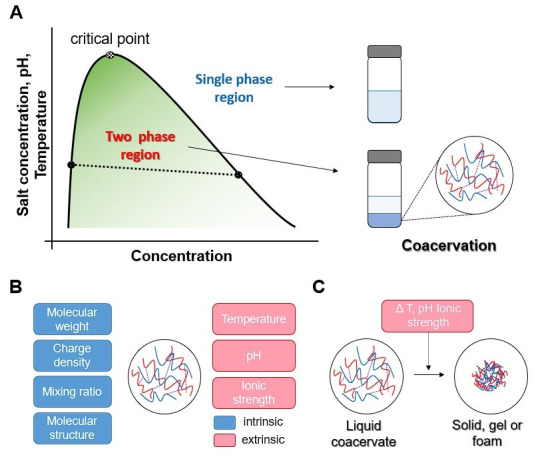
Illustration of (**A**) the range of LLPS of coacervation, (**B**) the intrinsic and extrinsic factors influencing the coacervation process, and (**C**) the phase transition of coacervates depending on temperature, pH, or ionic strength.

The process of coacervation is initiated by a delicate balance among the electrostatic interaction, hydrophobic interaction, hydrogen bonding, and van der Waals forces between distinct molecular species [41]. A disruption of this balance can lead to the disassembly of coacervation into a homogeneous single-phase solution, or alternatively, induce phase separation, leading to precipitation. Consequently, the physicochemical parameters of the system—including the molecular structure, ionic strength, pH, and molecular ratio of the components—play a significant role in the formation and stability of the coacervate phases [1]. Figure 2 shows the coacervation formation of the mechanisms and regulatory factors.

### 4.1. Molecular Structure

Molecular characteristics are a key factor influencing coacervation. The molecular structure is essential in determining the degree and properties of coacervation [1]. The arrangement, location, and density of the charges within the molecule can fluctuate based on its structure, thereby affecting the intermolecular interactions [42]. Variations in molecular size and topology (e.g., linear, branched, or secondary structures) alter the ion distribution, potentially influencing coacervate formation [43,44,45,46]. In addition, the chirality of a molecule can induce steric hindrance, resulting in unique aggregation behaviors that may cause precipitation instead of coacervation [47].

Perry et al. investigated the complex coacervation of PLys with PGlu by varying the polymer chirality [47]. The phase separation between oppositely charged PLys and PGlu was established by electrostatic interactions and hydrogen bonding. Coacervation occurred when at least one of the polypeptides was racemic, which disrupted the hydrogen bonding among the chains. In contrast, when both polypeptides possessed pure chirality, they formed solid precipitates with β-sheet structures due to steric hindrance [47].

Pramanik et al. demonstrated that histidine-rich peptides, specifically (GHGXY)_4_ (where X = Leu/Val/Pro), have a significant impact on coacervate formation [48]. The hydrophobicity of the amino acid residues exerted a substantial influence on the coacervation process. Leucine demonstrated enhanced coacervate stability and increased droplet sizes in comparison to those containing less hydrophobic residues such as valine. However, the impact of chirality (D- and L-amino acid variants) on coacervate characteristics did not show significant differences [48].

Vieregg et al. examined the formation of coacervates based on the structural characteristics of oligonucleotides [49]. Single-stranded DNA underwent coacervation with PLys due to its lower charge density. However, double-stranded DNA with its higher charge density led to the formation of solid precipitate [49].

### 4.2. pH

In the process of coacervation, pH serves as a key parameter by modulating the charge state of the molecules, thereby influencing electrostatic interactions and ultimately determining the occurrence and nature of phase separation. This is particularly significant for molecules containing weakly acidic or basic functional groups, as pH adjustments can effectively tune the degree of ionization. An appropriate pH environment enables the molecules to possess opposite charges, which is essential for the formation of coacervates [50,51].

Kayitmazer et al. investigated the formation of hyaluronic acid (HA) and chitosan (CS)-based coacervates as a function of pH [52]. Coacervation occurred within a pH range between the pKa values of HA and CS, where the ionic strength of both molecules increases. As the pH increased, the HA/CS system underwent a transition from a single-phase solution to coacervation, eventually resulting in a precipitated state [52].

Doshi et al. reported on the occurrence of pH-induced coacervation in legume protein mixtures [53]. The proteins remained soluble in the higher pH range, while the precipitation of protein particles occurred within the lower pH range. Within the intermediate pH range, coacervates were formed by the simple coacervation of proteins [53].

Jamshidian et al. investigated the complex coacervation of wheat germ protein and high methoxy pectin [54]. The pH of the solution was found to have a significant effect on the process of coacervation, with alterations to the surface charges of biomolecules. Changes in particle size and zeta potential were observed with varying pH levels. At pH_max_, the result exhibited the highest turbidity and the largest particle size [54].

### 4.3. Temperature

In contrast to specific systems that exhibit significant temperature sensitivity, the majority of coacervation systems demonstrate relatively weak temperature dependence. This thermo-responsive behavior is primarily governed by the intrinsic physicochemical properties of the individual macromolecule involved, such as its hydrophilicity, polyelectrolyte characteristics, and molecular conformation [1]. Macromolecules that exhibit thermo-responsive solubility can be classified based on two critical solution temperature behaviors: the Lower Critical Solution Temperature (LCST) and the Upper Critical Solution Temperature (UCST) [55,56]. In LCST systems, macromolecules are fully soluble at lower temperatures; however, upon heating, phase separation occurs. This behavior is predominantly driven by entropy. At low temperatures, macromolecule–solvent interactions are dominant. Elevated temperature enhances macromolecule–macromolecule interactions while diminishing macromolecule–solvent interactions. This transition facilitates phase separation. Conversely, UCST systems display the opposite trend. These systems remain a homogeneous mixture at elevated temperatures but undergo phase separation upon cooling. In this case, this behavior is predominantly driven by enthalpy [57,58]. Complex coacervation often exhibits phase behavior similar to that of LCST systems. Strong macromolecule–macromolecule interactions are present at higher temperatures, which weaken as the temperature decreases. The reduction in intramolecular and intermolecular interactions upon cooling leads to phase separation, given that the process is generally governed by entropic contributions. Coacervation under UCST conditions is rare, but has been reported in systems with strong enthalpic driving forces [59].

Fu et al. induced coacervation using five types of N-substituted polypeptoids (NNCAs) with different alkyl chain lengths: allyl, propargyl, butyl, hexyl, and octyl [60]. The polypeptoids exhibited both LCST and UCST systems. The phase transition temperatures were tunable within a range of 29–55 °C, depending on the chemical composition and the length of the alkyl side chains. This study demonstrated that the cloud point of the polypeptoids decreased with an increase in alkyl chain length. The cloud point decreased from 55.0 °C to 39.0 °C as the alkyl chain length increased from butyl to octyl [60].

The research conducted by Nie et al. presented the coacervation of a globular protein known as lipoate-protein ligase A (LplA) from *E. coli* [61]. LplA exhibited temperature-sensitive, reversible LCST coacervation. The coacervation began at approximately 14 °C, with significant structural changes occurring as the temperature increased. The emergence of larger particles between 12 °C and 16 °C in the protein solution specifically indicated the initial stages of the transition from monomeric LplA to oligomeric structures. This study also demonstrated that the coacervation of LplA could be selectively induced and reversed through the dissolution of the condensates by LplA’s natural substrate, lipoic acid, and its analogue, lipoamide [61].

### 4.4. Mixing Ratio

A pivotal factor in coacervation, especially in complex coacervation, is the charge stoichiometry between the positively and negatively charged biomolecules. Coacervation typically necessitates an electrically neutral state [1]. When synthetic polymers comprise solely one type of charged group, the calculation of charge stoichiometry to determine the mixing ratio is relatively straightforward [62]. However, the prediction of the mixing ratio is much more challenging for biomolecules like proteins that contain both positive and negative charges. The driving force for phase separation may arise not from the overall net charge but rather from the specific charge distributions on the molecular surface [63].

Wei et al. investigated the complex coacervation between theabrownin (TB) and whey protein isolate (WPI) depending on their mixing ratio. It was found that an intermediate pH induced complex coacervation with the strongest electrostatic interactions. The findings further demonstrated that the most robust electrostatic interaction was observed at a ratio of 10:1 of TB and WPI. The process of coacervation was mainly driven by electrostatic interactions, in conjunction with hydrogen bonding and hydrophobic interactions [64].

Cui et al. examined the coacervation of Antarctic krill protein isolate (AKPI) and GA. The coacervation process was conducted at a ratio of AKPI to GA of approximately 3:1 [65]. The electrostatic interaction and hydrogen bonding were identified as the main driving forces of coacervation. It was demonstrated that the mixing ratio not only influences the formation of coacervate but also has the potential to affect the ratio of wall to core [65].

Li et al. investigated the coacervation of chia seed gum (CSG) and WPI under WPI/CSG mass ratios (16:1–1:1, *w*/*w*). The stronger formation occurred at comparatively elevated mass ratios of 16:1, 8:1, and 4:1. With the rising proportion of WPI, there was a corresponding increase in the zeta potential value. The most pronounced electrostatic interaction was observed at the 4:1 ratio [66].

### 4.5. Ionic Strength

Ionic strength is determined by the concentration of ionic species present in the solution [67]. It regulates the electrostatic interactions among the charged components, thereby influencing the formation of coacervation [66,68]. Moderate ionic strength may facilitate coacervation; however, an excessive concentration of salt can inhibit this process. This concept is identified as the critical salt concentration [69,70]. Elevated ionic strength diminishes the electrostatic attractions between oppositely charged molecules, thereby significantly reducing their ability to undergo coacervation. However, coacervation exhibiting significant resistance to salt can still manifest LLPS and maintain stability under increased ionic strength [71,72].

Kayitmazer et al. demonstrated that HA and CS form a coacervate due to strong electrostatic interactions between them [52]. In their study, the coacervation was markedly suppressed at an ionic strength ≥ 1.5 M NaCl. At higher ionic strengths, the presence of additional ions in the solution screens the electrostatic interactions between the charged groups of HA and CS. This screening effect reduces the attractive forces that drive coacervation, making it more challenging for the polymers to come together and form coacervates. The study also demonstrated that, as ionic strength increases, there is a transition from coacervation to flocculation. This means that instead of forming a stable coacervate phase, the system may lead to the aggregation of particles (flocculation) due to the reduced electrostatic repulsion between them [52]. It is noteworthy that non-stoichiometric coacervation was observed when the zeta potential values were higher than zero, particularly at charge ratios less than 0.46, indicating that coacervation can happen even when the system is not electrically neutral, which is contrary to typical expectations [52].

Onuchic et al. investigated the effects of divalent cations (Mg^2+^, Ca^2+^, and Sr^2+^) on the formation of coacervates in a system consisting of an arginine-rich peptide (RP3) and polyU (RP3–polyU) [73]. They found that an elevated concentration of Mg^2+^ reduced the formation of coacervates. This result was consistent with the effects observed for monovalent cations (Na^+^), whereas divalent cations demonstrated a more pronounced influence. A comparable phenomenon was observed for other divalent cations, each demonstrating the reduced concentration thresholds necessary to initiate coacervation [73].

## 5. Biomolecule-Based Coacervation

### 5.1. Proteins

Proteins are well-known materials commonly used in coacervation processes. In recent years, protein-based coacervation has been increasingly studied not only in the field of life sciences but also in diverse applications such as food and cosmetics [66]. Protein-based coacervation primarily utilizes animal-derived charged proteins such as heparin, elastin, gelatin, tau, and BSA. More recently, protein extracts obtained from plants, such as soy, pea, and canola, have also been used in coacervation. This type of coacervation is mainly induced through electrostatic interactions between proteins and other proteins or polysaccharides [55,63,66,74,75,76,77,78]. Compared to synthetic polymers, protein-based coacervates offer advantages such as enhanced stability, biocompatibility, controlled release, permeability, and the retention of biological function.

Allahyartorkaman et al. conducted an investigation into the phosphorylation-induced simple coacervation of tau protein, as well as RNA-assisted complex coacervation [79]. The phosphorylation of tau protein triggered a simple coacervation upon a temperature transition from 4 °C to room temperature. The investigation revealed that, at concentrations below 2 μM, neither tau nor phosphorylated tau (p-tau) demonstrated coacervation. However, a binodal pattern of nucleation was observed when the concentration of p-tau was elevated to a range of 2 to 100 μM. The p-tau, in contrast to its non-phosphorylated counterpart, exhibited unique phase separation characteristics when subjected to different pH conditions. This indicates that phosphorylation is fundamental to the response of tau proteins to variations in pH, especially regarding their capacity for the formation of coacervates [79].

**Figure 3:**
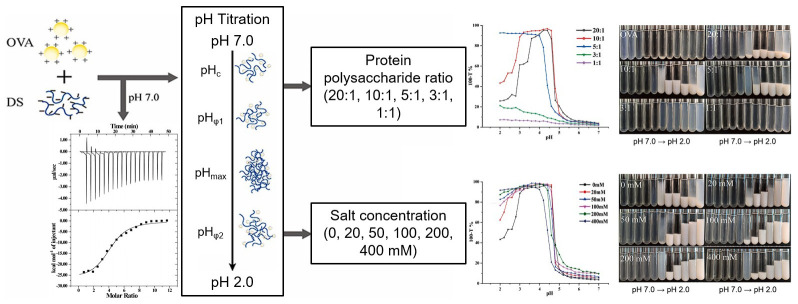
Schematic of the coacervation between ovalbumin and dextran sulfate under biopolymer ratio and salt concentration. The coacervation with OVA and dextran sulfate was induced by the protein/polysaccharide ration and salt concentration. (Adjusted from ref. [80]. Copyright © 2021 Elsevier Ltd.).

As shown in Figure 3, Liu et al. investigated the complex coacervation between ovalbumin (OVA) and dextran sulfate (DS) and their properties [80]. The research demonstrated that complex coacervation is significantly influenced by the ratio of OVA to DS. With an increase in the OVA/DS ratio, the critical pH values for coacervation were observed to shift towards higher values. The salt concentration also influenced coacervation. At low salt concentrations (≤100 mM), the solubility of OVA and DS was enhanced by reducing electrostatic repulsion. However, at elevated concentrations (≥100 mM), complex coacervation was inhibited. An increase in the concentration of salt ion within the solution resulted in a decrease in the critical pH, thereby reducing the electrostatic interaction. The thermodynamic parameters were employed to examine the stable coacervate formation between OVA and DS. The enthalpy changes suggested that the interaction between OVA and DS was exothermic, predominantly influenced by electrostatic attractions. The observed entropy change indicated that hydrophobic interactions play a role in coacervate formation, serving as an additional driving force for this process [80].

Archut et al. explored the interactions between various fractions of pea protein and pectin [81]. Soluble pea protein (SPP) was isolated from pea protein (PP) concentrate using isoelectric precipitation. Subsequently, the turbidity of SPP/pectin and PP/pectin coacervation was assessed to examine the interaction behavior. The study revealed that pea globulins played a substantial role in the increase in turbidity as a result of the protein’s simple coacervation. In contrast, the SPP did not affect the turbidity, indicating that these proteins primarily formed complexes with pectin without simple coacervation. A molecular weight distribution analysis further demonstrated that pea albumin and low-molecular-weight fractions below 20 kDA were essential for forming stable coacervates with pectin [81].

### 5.2. Nucleacids

Nucleic acids, such as negatively charged ssDNA or RNA [82], can induce coacervation through electrostatic interactions with oppositely charged cationic peptides or proteins. Nucleic acid-based coacervation is widely used in studies that mimic membraneless organelles such as stress granules and nucleoli [10,83]. Furthermore, it finds applications in targeted therapy and gene editing through the stable encapsulation of functional RNAs including mRNA and siRNA. Due to the sequence specificity of DNA/RNA, nucleic acid-based coacervation enables selective molecular recognition and binding. This process provides biocompatibility and allows for the precise regulation of biological responses [84,85,86].

Vieregg et al. investigated the phenomenon of coacervation involving oligonucleotides, specifically single-stranded and double-stranded DNA, in conjunction with cationic peptides [49]. Their findings revealed that coacervates were successfully formed between the peptides and single-stranded DNA, whereas no such interactions were observed with double-stranded DNA. At salt concentrations of 500–700 mM NaCl, single-stranded DNA and peptide complexes transitioned from precipitates to coacervates, while, at concentrations above 1 M, the formed coacervates dissociated. Under 300 mM NaCl conditions, 10 bp dsDNA exhibited a phase transition from precipitation to coacervation at 50 °C. Although most single-stranded sequences formed coacervates, certain purine-rich sequences induced precipitation even in the absence of hybridization [49].

**Figure 4:**
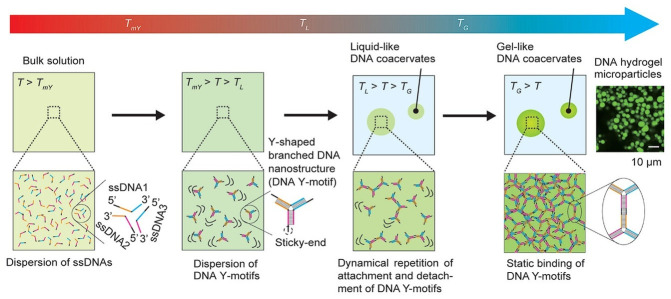
Schematic illustration of the formation of liquid- and gel-like DNA coacervates. When the solution temperature T > T_mY_, three kinds of single-stranded DNAs (ssDNA1, ssDNA2, and ssDNA3) are dispersed in the bulk solutions; when T_mY_ > T > T_L_, the three ssDNAs form a DNA Y-motif with self-complementary sticky ends; when T_L_ > T > T_G_, liquid-like coacervates are formed by the dynamical repetition of attachment and detachment of the sticky ends of the DNA Y-motifs; when T_G_ > T, gel-like DNA coacervates are formed by the static binding of the DNA Y-motifs via their sticky ends. T_mY_ is the melting temperature of the Y-motif stem. T_L_ is the formation temperature of liquid-like DNA coacervates. T_G_ is the formation temperature of gel-like DNA coacervates. (Adjusted from ref. [87]. Copyright 2024, the Authors, published open access by *Advanced Materials Interfaces* under the terms of the Creative Commons CC BY License).

Morita et al. showed DNA-based coacervation from branched DNA nanostructures [87]. The results demonstrated that the physical properties of these structures can be controlled by manipulating factors such as cooling rates and DNA concentrations (Figure 4). The process of coacervation of three single-stranded DNAs (ssDNA1, ssDNA2, and ssDNA3), which are designed to form Y-motifs, was examined under varying temperature conditions. When the solution temperature T exceeds TmY (T_mY_ = melting temperature of the Y-motif stem), the ssDNAs are completely dissociated. Under conditions where TmY > T > TL (T_L_: temperature for the formation of liquid-like DNA coacervates), dispersed DNA Y-motifs are formed. In the range of TL > T > TG (T_G_: temperature for the formation of gel-like DNA coacervates), the Y-motifs dynamically bind and unbind via their sticky ends, leading to the formation of liquid-like DNA coacervates. When T < T_G_, the Y-motifs form gel-like DNA coacervates through static binding via their sticky ends. Under the experimental conditions, T_mY_ was 75 °C and the measured T_L_ and T_G_ were approximately 64 °C and 35 °C, respectively [87].

In a study, van Haren et al. investigated how coacervates (biomolecular condensates) are formed through the enzymatic deacetylation of peptides in the presence of DNA [88]. They found that coacervation did not occur when only DNA and peptides were present. However, when deacetylation was induced using the enzyme SIRT3, the coacervation between DNA and peptides was successfully triggered under the same conditions. At low concentrations of the enzyme, the weak interactions between DNA and peptides led to the formation of gel-like coacervates. In contrast, at higher enzyme concentrations, stronger interactions were promoted, resulting in a transition to liquid-like coacervates [88].

### 5.3. Peptides

Peptides possess a simple backbone structure and various functional groups on their side chains, contributing to molecular self-assembly [89,90]. Hydrophobic interactions and hydrogen bonding within the peptide chains promote the formation of special secondary structures such as α-helices and β-sheets, which can further organize into unique architectures like fibers, nanotubes, and nanowires [91,92,93]. Based on these structural advantages, peptides are highly responsive to external stimuli. In particular, due to their specific sequences, peptides can not only undergo self-assembly but also interact with other molecules such as nucleic acids and polysaccharides to induce coacervation [94,95].

Gulão et al. investigated the complex coacervation based on polypeptide leucine with GA. The optimal pH for coacervation was found to be below 4.0 with increased turbidity and particle size at this pH range. When the concentration of polypeptide leucine and GA was 0.2% and 0.03% and there was a no salt condition, the coacervation had higher turbidity and the highest particle size. The elastic behavior of coacervates also increased under the same conditions [39].

The coacervation based on elastin-like polypeptides (ELPs) was investigated by Fisher et al. [96]. The ELPs were engineered to include either positively charged residues (e.g., K30, KQ30) or negatively charged residues (D/E30, D/EQ30) to enable their use in complex coacervation. The charge balance was found to be a critical factor for effective coacervation, with the most pronounced turbidity and material yield observed when the mixture contained a slight excess of positively charged ELPs. Coacervation was highly sensitive to salt concentration; for instance, the coacervates composed of K30 and DE30 completely disappeared upon the addition of 50 mM NaCl. Similarly, for ELPs containing Q and fewer charged residues, coacervation was suppressed even at 30 mM NaCl. The formation of coacervates was analyzed through turbidity measurements and optical microscopy observations [96].

**Figure 5:**
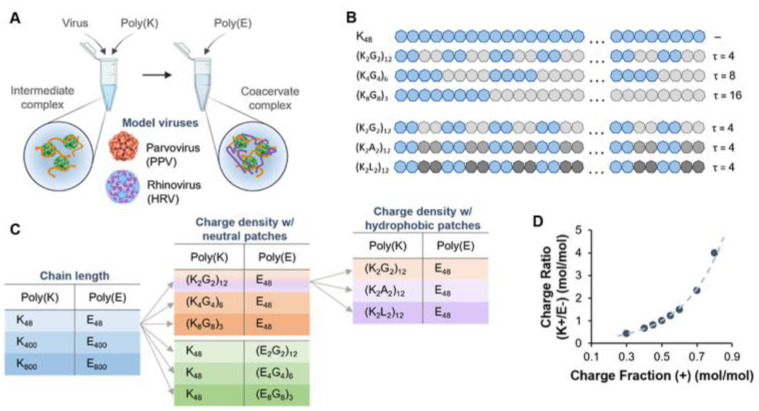
Coacervate design using sequence-defined block-*co*-polypeptides. (**A**) Schematic depiction of virus-containing coacervate formulation. (**B**) Example depiction of the variations in polypeptide charge density and hydrophobicity of the cationic lysine (K)-containing polymers. Charge blockiness is defined by the parameter τ, while hydrophobicity is indicated by the color of the gray blocks as the neutral amino acid spacers go from glycine to alanine to leucine. (**C**) Experimental design matrix to study the effect of polypeptide characteristics such as charge patterning and hydrophobicity on virus encapsulation. (**D**) Plot of the charge ratio (K+/E−) as a function of the total cationic charge fraction from the polypeptides present in the system. (Reused from ref. [97]. Copyright 2023, the authors published open access by *Biomacromolecules* under the terms of the CC-BY-NC-ND 4.0).

Joshi et al. investigated the complex coacervation with polypeptides to encapsulate nonenveloped viruses, specifically porcine parvovirus (PPV) and human rhinovirus (HRV) [97] (Figure 5). The peptide properties, such as peptide chemistry, chain length, charge patterning, and hydrophobicity, effected the virus incorporation. Virus encapsulation is primarily driven by electrostatic interactions, with the optimal charge fraction (i.e., the ratio of cationic monomers) typically falling between 0.4 and 0.6. When analyzing coacervation according to polypeptide chain length—short (N = 48), medium (N = 400), and long (N = 800)—the long chains (N = 800) shifted the optimal coacervation condition toward a lower cationic charge fraction. In the case of polypeptides with alternating charged and neutral residues (e.g., (K2G2)12), charge clustering was shown to influence encapsulation efficiency. Regarding the charge density, the polypeptides with a high charge density demonstrated the highest encapsulation efficiency, while introducing neutral residues to reduce the charge density led to decreased efficiency. Increasing the hydrophobicity of the peptides (e.g., K2G2→K2A2→K2L2) affected PPV encapsulation moderately, whereas HRV encapsulation was significantly enhanced when leucine was incorporated into the peptide sequence [97].

### 5.4. Polysaccharides

Polysaccharides are natural polymers with high biocompatibility, biodegradability, and functional groups, making them widely studied for coacervation in various biological fields. Commonly used polysaccharides include alginate, HA, GA, pectin, and dextran. Due to their chemical properties, polysaccharides typically carry a negative charge and form coacervates through electrostatic interactions with positively charged molecules such as proteins and peptides [32,63,98,99]. Being naturally derived, polysaccharides exhibit low toxicity and excellent biocompatibility, and they can stably protect encapsulated biomolecules from external environments. In addition, they allow for reversible phase separation in response to changes in pH or ionic strength [100].

Li et al. investigated the interaction involved in coacervation between different types of polysaccharides (CS and carboxymethyl cellulose) and soy protein isolate under various conditions. The coacervate with carboxymethyl cellulose (CMC) and CS and soy protein isolate (SPI) exhibited gel-type rheological behaviors (elastic modulus (G’) values = 1000 Pa). The addition of salt ions (50 mM NaCl) decreased the elastic and viscous modulus (G′ and G′′) of the SPI/CS and SPI/CMC coacervates from 1000 Pa to 100 Pa. The peak viscosity of SPI simple coacervates occurred at a pH near its isoelectric point of 4.5. However, the coacervate of SPI with CS and CMC demonstrated higher viscosity than the coacervates of SPI alone [101].

Yuan et al. investigated complex coacervation with SPI and CS for the microencapsulation of algal oil in a separate study on coacervation utilizing CS [102]. The optimal pH and CS/SPI ratio were 6.0 and 0.125 g/g, respectively. The interaction between CS and SPI showed a strong affinity (Ka = 4.16 × 10^5^ ± 0.09 × 10^5^ M^−1^). The CS/SPI coacervates exhibited higher encapsulation efficiency (97.36 ± 1.16) and improved oxidative stability compared to the simple coacervation of SPI [102].

**Figure 6:**
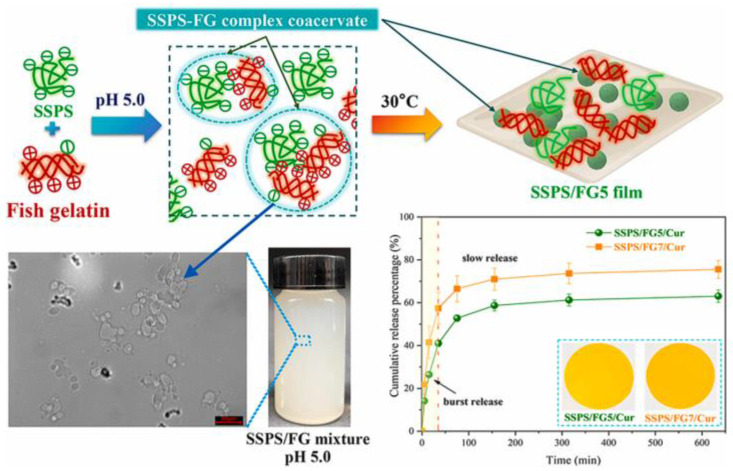
Polysaccharide-based coacervation with soy soluble soybean polysaccharide (SSPS) and fish gelatin. The coacervates were induced at a pH of 5.0 and coated for film. The curcumin-encapsulated coacervate film exhibited both burst release and slow release. (Reused from ref. [103]. Copyright © 2024 Elsevier Ltd.).

As shown in Figure 6, Wang et al. developed a blend film of soluble soybean polysaccharide (SSPS) and fish gelatin (FG) through complex coacervation [103]. The coacervates were fabricated using an SSPS/FG mass ratio of 1:3. The electrostatic interaction between SSPS and FG at a pH of 5.0 promoted complex coacervation in the film-forming solution. The coacervation with SSPS and FG exhibited increased surface roughness, enhanced thermal stability, and improved water vapor barrier properties. The SSPS/FG coacervate delayed the release of curcumin from the films into food simulants during a release test [103].

## 6. Application of Biomolecule-Based Coacervation

Coacervation is a liquid–liquid phase separation process that enables the formation of concentrated biomolecular droplets. In contrast to coacervation using synthetic polymers with defined charges, coacervation with natural biomacromolecules such as proteins, polysaccharides, nucleic acids, and polypeptides provides superior biocompatibility and biodegradability [104,105,106]. Biomolecule-based coacervates are being actively explored for their multifunctional roles in biomedical and industrial applications [107,108,109]. They have been utilized in the encapsulation of bioactive compounds, the stabilization of vaccines, and the formulation of functional foods [110,111,112]. In particular, their responsiveness to environmental stimuli such as pH and temperature makes them ideal for site-specific release [109,113]. Recently, numerous studies have explored the application of coacervation in underwater adhesion and tissue engineering [114,115]. Furthermore, coacervates can mimic intracellular compartments, contributing to research on the origin of life and the development of artificial cells [116]. Table 1 presents the recent trends in coacervation for the biotechnology and biomedical fields.

**Table 1:** Recent study of coacervation for biotechnology and biomedical applications.

Bioapplication	Biomolecule Component	Cargo	Study Objective	Ref.
Encapsulation	SA/CS	Walnut oil	Improved higher loading capacity and oxidation stability	[117]
Encapsulation	GA/CS or GA/trehalose/CS	*Lacticaseibacillus rhamnosus*	Promoted the stability of probiotic bacteria	[118]
Encapsulation	Gelatin/chia mucilage	Oregano essential oil	Developed encapsulation system for spray drying and improved encapsulation efficiency	[119]
Encapsulation	Zein/CS	Resveratrol	Improved encapsulation efficiency and dispersion stability via atmospheric cold plasma	[120]
Encapsulation	Plum seed protein isolate/polysaccharides	Essential oils	Enhanced stability, storage, emulsification, and encapsulation	[121]
Encapsulation	Zein–gallic acid/CS	Gallic acid	Induced structural modifications of encapsulation and enhanced thermal stability	[122]
Encapsulation	SPI/CS	Deer oil	Enhanced the stability of encapsulation against oxidative stress and encapsulation efficiency.	[123]
Encapsulation	β-conglycinin/lysozyme	Curcumin	Improved encapsulation efficiency, loading capacity, and stability against light and heat treatment	[124]
Encapsulation	GA/Krill protein isolate	Antarctic Krill oil	Developed stable and biocompatible encapsulation for oil	[65]
Encapsulation	WPI/GA	Tuna oil	Enhanced oxidative stability and made encapsulation more thermosensitively suitable	[125]
Encapsulation	WPI/flaxseed gum/monodiglyceride fatty acids	Resveratrol	Improved stability, encapsulation efficiency, and sustained antioxidant ability	[126]
Delivery platform	Cholesterol-modified DNA/histone	Virus particle, mRNA, cytokines, peptides	Enhanced the stability of the delivery vehicle biological agents	[127]
Delivery platform	Fungal CS/GA	α-tocopherol	Promoted stable and easy-to-prepare encapsulation materials for harsh conditions	[128]
Delivery platform	LMWG or HMWG/SA	miRNA-497	Developed a biocompatible delivery system to enhance cellular uptake and stability	[129]
Delivery platform	Heparin GAG/tyrosine- and arginine-based peptide	Tannic acid	Developed a stable and permeable delivery system that released drugs in response to biological triggers	[130]
Delivery platform	Dextran graft copolymer	DNA	Enhanced release capabilities and transfection	[131]
Delivery platform	Ellagic acid/casein	Ellagic acid	Enhanced oral absorption and improved solubility	[132]
Delivery platform	Peptide	pDNA, mRNA/sgRNA, RNP	Developed a redox-triggered delivery vehicle for CRISRP-Cas9 genome editing	[133]
Delivery platform	Peptide	siRNA, pDNA, mRNA	Developed a pH-responsive delivery nanocarrier for cancer therapy	[134]
Delivery platform	Single-stranded DNA/PLL	Emamectin benzoates	Improved loading capacities and stability against photodegradation	[135]
Delivery platform	PEAD/heparin	BMP-2	Developed a protein delivery platform with enhanced colloidal stability	[136]
Tissue engineering	CMC/gelatin	-	Developed 3D extrusion printing hydrogel with higher printing fidelity and without any discontinuities during the printing process	[137]
Tissue engineering	Gleatin/GA/CMC	β-carotene	Improved thermal, pH, and ionic strength stability and evaluated its potential applications in surimi	[138]
Tissue engineering	HA/CS	Rat BMSCs	Developed chondro-inductivity scaffold for encapsulating BMSCs	[139]
Tissue engineering	Gelatin/QHECE	Glucose oxidase	Developed glucose-responsive microneedle loaded with glucose oxidase and enhanced drug release	[140]
Tissue engineering	LMWC/HA or HMWC/HA	-	Developed biocompatible hydrogel with shape adaptability and enhanced wet adhesion	[141]
Tissue engineering	SPI/chelator-soluble pectin	β-conglycinin, glycinin	Developed food inks for 3D printing with enhanced particle distribution and mechanical properties	[142]
Tissue engineering	Theabrownin/whey protein isolate	-	Developed coacervate for modulating energy metabolism and mitochondrial apoptosis to strengthen muscle cells	[64]
Tissue engineering	ApoEVs/GelMA or curcumin/CMCS/GelMA	Apoptotic extracellular vesicles, curcumin	Developed multifunctional 3D-printed scaffold for enhancing skin regeneration and promoting antibacterial activity and ROS scavenging activity	[143]
Tissue engineering	PEAD/heparin	Cargo IGF-1	Enhanced bioactivity of cargo IGF-1 and sustained release to embed in cartilage regeneration hydrogel	[144]
Tissue engineering	Egg yolk/CMC	β-carotene	Enhanced stability of interfacial layer and structural strength	[145]
Adhesive technology	Methacrylated LMWC/HA	-	Developed coacervate with enhanced wet tissue adhesion and tunable properties	[146]
Adhesive technology	CS/HA	-	Enhanced underwater adhesion strength against salt switch conditions and promoted antibacterial properties	[147]
Adhesive technology	Peptide/polyoxometalate	-	Developed injectable, self-solidifying underwater adhesion and enhanced its properties	[148]
Adhesive technology	Tyramine-conjugated alginate/RGD peptide	Calcium phosphate	Developed a photo-mineralized hydrogel and enhanced adhesiveness and bioactivity of bones	[149]
Cellular mimicking	PDDA/ATP	Dextran	Developed a demembranization system that reconfigures in response to biological signals with enhanced permeability	[150]
Cellular mimicking	RNA/peptide	RNA	Developed fuel-dependent RNA-containing coacervation that mimics membraneless organelles	[83]
Cellular mimicking	Dextran/polyaspartic acid	DNA, enzymes	Developed a biomimetic platform capable of biomacromolecule segregation, reaction control, and morphological reconfiguration	[151]
Cellular mimicking	PEG/dextran	DNA	Developed compartmentalized artificial cell structures to mimic and investigate spatiotemporal control mechanisms	[152]

### 6.1. Encapsulation

Encapsulation enables the isolation and protection of sensitive molecules by surrounding them with a phase-separated environment. The ability of coacervation to accommodate so-called client molecules is one of its most intriguing features [111,112,153]. Client molecules, such as proteins and other small compounds, can be encapsulated during the coacervate phase to study the biological processes in a more precise and controlled environment [154,155]. In particular, the presence of coacervates within an aqueous solution has been observed to exhibit remarkably low interfacial energy sufficiency, thereby enabling the absorption of a wide range of substances [156,157]. Encapsulation can be achieved through two distinct mechanisms. The first mechanism involves incorporating the cargo as a component of the coacervate system. The second mechanism occurs through specific interactions and preferential partitioning [158,159,160,161]. Cargo encapsulation via coacervation has been demonstrated to stabilize and protect the cargo within an aqueous environment, while also allowing for controlled release in response to external stimuli or environmental changes through the dissociation of the coacervation [162,163]. Coacervation-based encapsulation offers two key advantages: (1) the ability to perform encapsulation entirely in an aqueous environment, and (2) the potential to significantly concentrate the target molecule in the macromolecule-rich coacervate phase compared to the initial solution [40,164].

Barajas-Álvarez et al. demonstrated coacervation in GA/CS or GA/trehalose/CS crosslinking with tripolyphosphate to encapsulate *Lacticaseibacillus rhamnosus* probiotic [118]. This study evaluated the encapsulation efficiency, physicochemical properties, and probiotic survival under storage conditions and simulated gastrointestinal fluids. Furthermore, crosslinking with tripolyphosphate improved the encapsulation efficiency after the drying process. The probiotics encapsulated within coacervate showed a higher level of short-chain fatty acid production in comparison to the non-encapsulated probiotics [118].

**Figure 7:**
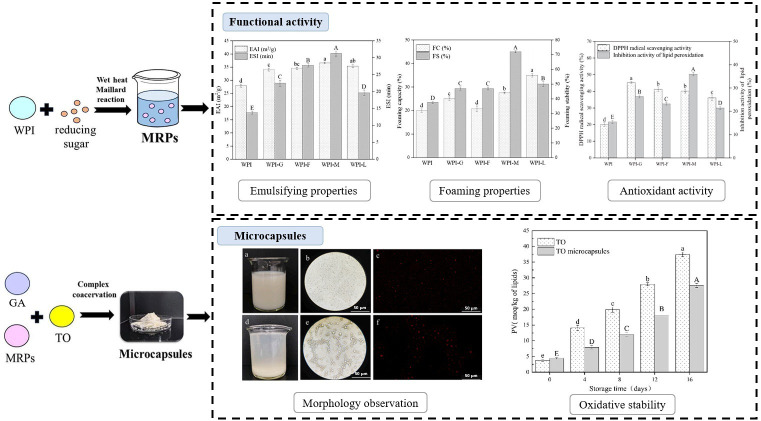
Schematic illustration of coacervation with whey protein isolate (WPI) and reducing sugar for encapsulating based on Maillard reaction. Functional activity (emulsifying properties, foaming properties, and antioxidant activity) of coacervates between WPI and different kinds of polysaccharides were analyzed. Tuna oil (TO) was encapsulated by complex coacervation with gum arabic (GA) and Maillard reaction products (MRPs). Different lowercase and uppercase letters indicate statistically significant differences among groups. Lowercase letters represent significant differences within each analytical parameter across different times or conditions, while uppercase letters indicate significant differences among different analytical parameters under the same condition (*p* < 0.05). (Reused from ref. [125]. Copyright © 2025 Elsevier Ltd.).

As shown in Figure 7, Wang et al. demonstrated the encapsulation of tuna oil (TO) through the process of coacervation with WPI and various reducing sugars, including glucose, fructose, maltose, and lactose [125]. The coacervates fabricated with WPI, GA, and maltose demonstrated an enhanced storage stability of TO. The encapsulation efficiency of WPI/maltose/GA was found to be 87.41%, which is higher than the encapsulation efficiency of WPI self-coacervation. Microencapsulated-protected TO had a 35.78% lower peroxide value than free TO after 16 days of accelerated oxidation at 55 °C [125].

Research has also been conducted on the modification of coacervation components to enhance their functionality. Xue et al. [121] modified plum seed protein isolate (PSPI) by enzymatic hydrolysis using alcalase, pepsin, and flavourzyme to conjugate with polyphenols such as atechin, curcumin, and proanthocyanidin. The modified PSPI was then used to form coacervates with various polysaccharides, and their structural and functional properties were evaluated. As a result, the modified PSPI/polysaccharide coacervates effectively maintained the stability of essential oils and exhibited improved emulsifying and encapsulating properties [121].

Despite the broad application of coacervation in the encapsulation of various hydrophobic and hydrophilic cargos, there is still a lack of systematic studies examining how the molecular characteristics of small molecules influence the encapsulation mechanisms, phase behavior, and material properties [165]. The work of Zhao et al. demonstrated that the partitioning of small molecule dyes into various polymer-based coacervates is primarily determined by their charge and hydrophobicity [166,167].

On the other hand, while coacervation enables the stable encapsulation of proteins, not all target proteins possess strong charges, which is an ongoing challenge in the field. To address this, Obermeyer et al. proposed a solution to the problem of binary protein–polyelectrolyte coacervates. Using conjugation chemistry, they artificially enhanced the native charge of proteins by modifying them with succinic anhydride, thereby generating supercharged proteins. Their study further demonstrated that a relatively low degree of supercharging—with a negative-to-positive charge ratio of approximately 1.1 to 1.4—was sufficient to induce coacervation with cationic polymers [168].

### 6.2. Delivery Platform

The use of coacervation for biomedical applications is based on the advantages of complex coacervation for encapsulation. Recently, coacervation systems using naturally derived polymers such as heparin, CS, and alginate have been investigated as delivery platforms [169,170]. For example, coacervates composed of CS and nucleic acids have been developed to deliver DNA and RNA to mammalian cells [171,172]. In contrast, recent research has demonstrated that peptide-based coacervation can be employed to deliver functional nucleic acids for gene therapy or cancer therapy [133,134].

Delaporte et al. investigated animal-free coacervation. The study used fungal CS to induce coacervation with GA. While the electrostatic interactions of fungal CS/GA coacervation decreased in different solvents (isohexadecance, ethylhexyl stearate, and ethanol), the wetting properties of the coacervates were improved. The encapsulation efficiency of α-tocopherol by coacervates was 82.6% at a 1:1 material-to-active mass ratio [128].

**Figure 8:**
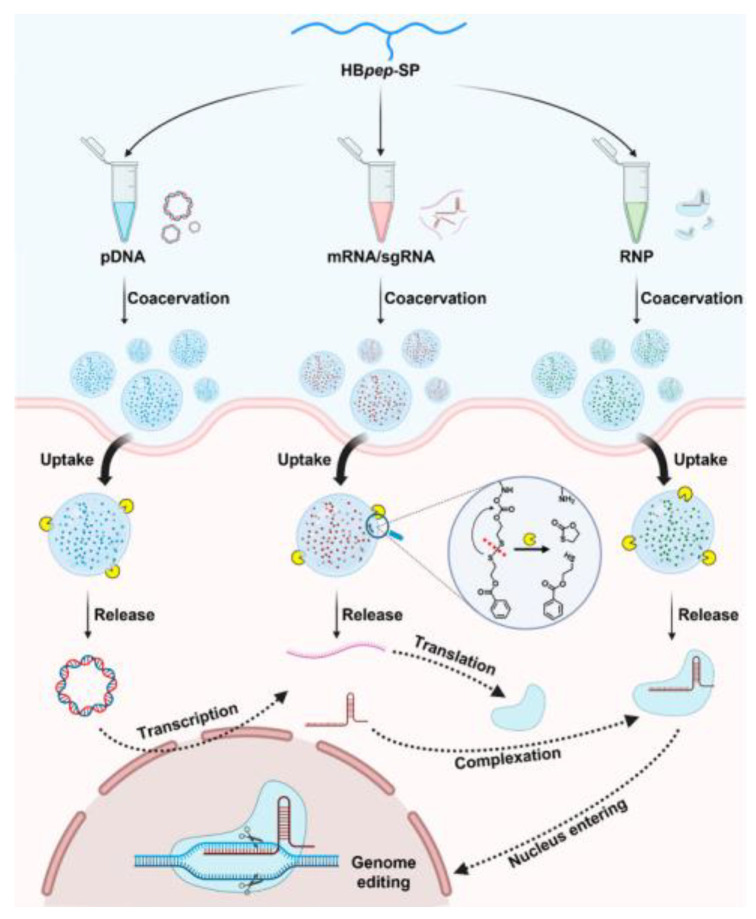
Schematic illustration of the universal delivery of CRISPR/Cas9 genome editing machineries mediated by HB*pep*-SP coacervates. Three types of CRISPR/Cas9 genome editing machineries including pDNA, mRNA/sgRNA, and Cas9 ribonucleoprotein (RNP) can be readily recruited during the LLPS of the HB*pep*-SP peptide. The cargo-loaded coacervates are internalized by the cell and then reduced by glutathione (the yellow spheres) in the cytosol, triggering cleavage of the side chain modification and disassembly of the cargo-loaded coacervates. The Cas9 RNP, which is directly released from coacervates or produced by the transcription and translation of pDNA and mRNA, enters the nucleus and induces double-strand breaks on the genomic DNA. (Reused from ref. [133]. Copyright © 2023 American Chemical Society.).

Sun et al. used peptide coacervates for CRISPR/Cas9 genome editing (Figure 8). Peptide coacervation was induced in 20 mM phosphate buffer (pH 6.5, ionic strength 100 mM) and successfully encapsulated pDNA, mRNA, and Cas9 nuclease. The delivered cargo resulted in improved transfection and gene editing efficiency compared to conventional reagents [133].

Wang et al. investigated pH-responsive coacervates with peptides for nucleic acid delivery that respond to the different physiological pH conditions of various microenvironments such as tumors, lysosomes, and the bloodstream. The peptide coacervates delivered siRNA, pDNA, and mRNA into cancer cells [134].

In addition, Li et al. reported the application of coacervation with zein and CS for the sustained release of curcumin [173]. Lee et al. loaded injectable hydrogel coacervates with bortezomib, an anticancer drug [174]. Huei et al. applied iron (Fe)-crosslinked CMC-based coacervates for the controlled release of ibuprofen [175]. The strategy of using coacervates as a delivery platform has been shown to be effective in various studies. This approach utilizes the flexible and modular nature of charge-driven coacervate formation to address the challenges of (1) the protection and/or isolation of the drug, (2) the targeted distribution and absorption into specific cells or tissues, and (3) the controlled release of the drug over time.

### 6.3. Tissue Engineering

Tissue engineering aims to restore, maintain, improve, or replace tissues and organs using a combination of cells, scaffolds, and growth factors. These scaffolds are designed to mimic the extracellular matrix (ECM) and provide physical support for cell adhesion and proliferation [176,177]. Scaffold fabrication has traditionally relied on the gelation of precursor materials through chemical crosslinking methods such as photo-polymerization, enzymatic reactions, and click chemistry. However, such scaffolds often form simple, homogeneous gel structures, making it difficult to implement microscale patterning. Because conventional scaffold design focuses primarily on external cellular support, it does not adequately mimic the intracellular environment. As a strategy to enhance the functional properties of conventional scaffolds, recent studies have begun to incorporate coacervation [178]. Scaffolds produced by coacervation can form membraneless compartments that resemble intracellular organelles, thereby enabling the mimicry of intracellular microenvironments. Such coacervate-based scaffolds allow for the selective concentration of bioactive molecules, such as functional RNAs or growth factors, and help to maintain their bioactivity. Through electrostatic interactions, these encapsulated functional biomolecules can be released in a stimulus-responsive manner (e.g., in response to pH or ATP).

For example, Karabıyık Acar et al. developed a coacervate-based scaffold using HA and CS for cartilage repair. The HA/CS coacervates effectively encapsulated bone marrow mesenchymal stem cells (BMSCs) and demonstrated their efficacy in vitro. Chondrogenic markers such as ACAN, COL2A1, and SOX9 were upregulated following chondrogenic induction. Notably, even in the absence of chondrogenic stimuli, the encapsulated cells within the coacervates exhibited increased expression of cartilage-related markers, indicating their intrinsic chondro-inductive potential [137].

In another line of research, advances in tissue engineering have also led to progress in 3D printing-based scaffold fabrication for precise microenvironmental control. Gharanjig et al. proposed an extrudable hydrogel based on CMC/gelatin complex coacervation. Their study analyzed the rheological properties required for 3D printing and demonstrated that a CMC-to-gelatin ratio of 1:15 resulted in the highest print fidelity, with no observed discontinuities during extrusion [137].

**Figure 9:**
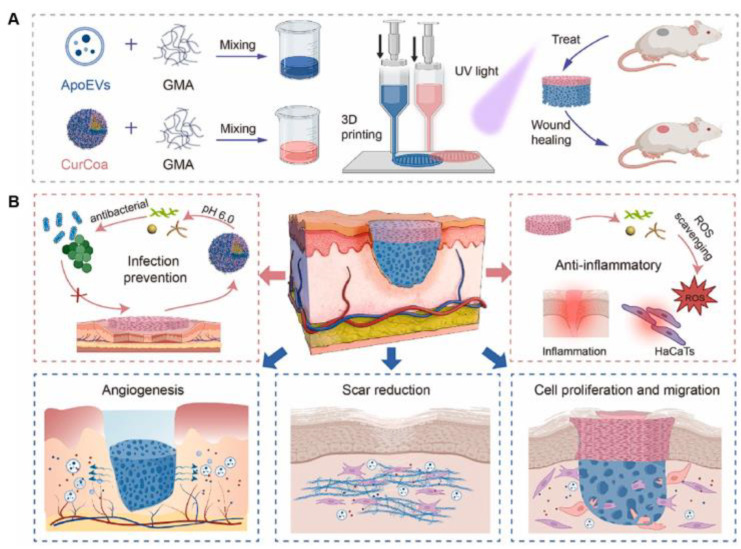
(**A**) Schematic illustration of ApoEVs/GelMA and CurCoa/GelMA coacervation for 3D-printed tissue engineering. (**B**) Scaffold based on ApoEVs/GelMA and CurCoa/GelMA coacervates showed various properties such as infection prevention, anti-inflammatory, angiogenesis, scar reduction, and cell proliferation and migration. (Reused from ref. [143]. Copyright © 2025 Elsevier Ltd.).

Jiang et al. developed a unique 3D-printed bilayer multifunctional scaffold incorporating regenerative apoptotic extracellular vesicles (ApoEVs) and antibacterial coacervates that showed strong potential for full thickness wound healing and revealed the underlying healing mechanisms (Figure 9). The upper layer, mimicking the epidermis, formed a dense structure to protect the wound from mechanical impact and pressure. The lower layer, mimicking the dermis, contained larger pores to promote cell migration and proliferation. Furthermore, the top layer included pH-responsive curcumin coacervates that exhibited antibacterial and reactive oxygen species (ROS) scavenging properties. The lower layer supported the sustained release of ApoEVs and enhanced fibroblast proliferation and migration, as well as angiogenesis in the endothelial cells. In vivo experiments confirmed that the scaffold accelerated wound healing and reduced scar tissue formation. Moreover, the RNA sequencing analysis revealed the molecular mechanisms by which the bilayer scaffold contributed to wound repair [143].

### 6.4. Adhesive Technology

The use of coacervation as a strategy for the development of surgical adhesives has recently attracted growing research interest [115,179]. This process has been observed in natural biological mechanisms, where various species use underwater adhesives to attach to different surfaces [180]. Representative examples include the sandcastle worm and mussels, in which coacervate-based adhesive systems were first identified [115,181,182]. In these organisms, coacervation has been found to play a critical role in the formation of adhesive materials under wet conditions [183]. Coacervation based on biomacromolecules has shown great potential as a wet adhesive, exhibiting excellent performance even in hydrated environments [184].

Recently, research has continued to focus on the development of underwater adhesives based on the immiscibility of coacervation in water and the strong adhesive properties of mussel adhesive proteins (MAPs) [185]. Lim et al. developed a MAP-based encapsulated coacervate system and applied it as a smart tissue adhesive capable of drug delivery. In this study, cationic recombinant hybrid MAPs (fp-131 or fp-151) and anionic HA were complexed by coacervation to form an adhesive. On aluminum surfaces, the bulk adhesive strength of this coacervate was more than twice as strong as that of the protein alone [186].

Furthermore, in a study by Yun et al. [149], an injectable adhesive hydrogel was prepared by coacervation using tyramine-conjugated alginate and RGD peptide-fused mussel adhesive proteins. Based on this coacervate hydrogel platform, a photoreactive agent, calcium ions, and phosphonodiol were incorporated to provide dual functionality: photocrosslinking and the formation of amorphous calcium phosphate, both activated by visible light irradiation. In in vivo experiments using a rat femoral tunnel defect model, the developed adhesive hydrogel demonstrated easy applicability to irregular defect sites, rapid bone regeneration without the need for bone grafting, and excellent integration with the surrounding tissue [149]

Several additional studies have been conducted on mussel adhesive protein (MAP)-based adhesives. Cha et al. produced rfp-1 MAP (AKP-SYPPTYK) using recombinant protein expression techniques. This MAP was used to form hydrogels by coordinating crosslinking with Fe^3+^ ions or covalent crosslinking with NaIO_4_ [187]. Lu et al. designed a hybrid molecular adhesive by combining CsgA protein, an amyloid-based adhesive protein from *E. coli*, with mussel adhesive proteins [188].

**Figure 10:**
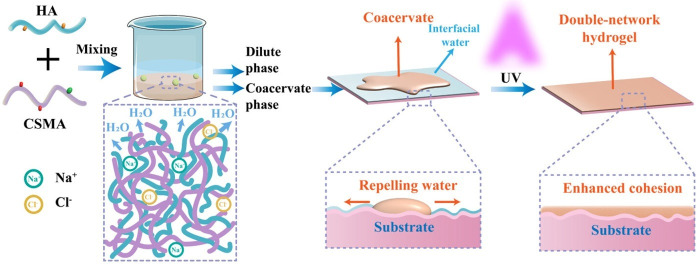
Schematic illustration of coacervation for adhesive technology: HA and low-molecular-weight methacrylated chitosan-formed coacervation. The coacervate was coated on the interfacial water of the substrate and treated by UV to form double-network hydrogel. Double-network hydrogel enhanced the cohesion of the substrate compared to a blank substrate. (Reused from ref. [146]. Copyright © 2025 American Chemical Society).

In addition to mussel protein-based adhesives, research has also explored polysaccharide-based adhesion technologies. Deng et al. proposed a wet tissue adhesive based on the coacervates formed from low-molecular-weight methacrylated chitosan (CSMA) and HA (Figure 10). The fabricated coacervates can be applied to wet tissue surfaces and photo-crosslinked to form in situ double-network hydrogels, which enhances cohesion and ensures long-lasting adhesion. After immersion in PBS for 24 h, the hydrogel burst pressure increased to approximately 623 mmHg due to dynamic bond reorganization and low swelling ability [146].

Despite these advances in coacervation-based research, a complete tissue adhesive that is non-toxic, biocompatible, biodegradable, user-friendly, scalable, and capable of mimicking natural adhesion has yet to be developed. Future research needs to investigate how the chemical structure of the polymer backbone, hydrophilicity/hydrophobicity, combinations of amino acids, and charged side groups influence the adhesive strength. In addition, the application of tissue adhesives should expand beyond basic tissue bonding to include drug delivery, tissue grafting, wound healing, and tissue regeneration, providing new opportunities for biomedical advancement.

### 6.5. Cellular Mimicking

Coacervation occurs when oppositely charged polymers interact in water, resulting in LLPS and the formation of highly concentrated liquid droplets [189]. Although this phase separation is similar to the behavior of oil and water, the droplets represent a distinct aqueous phase within the water, often referred to as a “second liquid” [190]. In the past, Oparin observed these properties of coacervates and proposed that such structures may have functioned as protocells in the prebiotic era, prior to the emergence of life [191]. However, this hypothesis was largely dismissed at the time because coacervates lack membranes, whereas real cells are clearly defined by their membrane structures. Recently, this idea has regained significant attention due to the discovery of membraneless organelles such as the nucleoli [192], P granules, and stress granules [193,194,195]. These findings have revitalized research in the field, supporting the design of synthetic cells and artificial organelles and extending the applications of coacervation to areas such as origin-of-life studies, neurodegenerative diseases, cellular compartmentalization, and protein-based drug delivery [194,196].

For example, Jia et al. generated polyester coacervates based on prebiotically accessible α-hydroxy acids. The formation of these coacervates demonstrated their ability to partition proteins and RNA in a manner consistent with origin-of-life conditions [197]. Longo et al. further demonstrated that coacervation can facilitate the conversion of abiotic ornithine residues into arginine, thereby enabling the synthesis of proteins capable of binding dsDNA [198].

In another study, Donau et al. proposed a coacervate-based model to understand the behavior of membraneless organelles. In their study, they prepared and used active coacervates containing RNA that were driven by ATP-dependent reactions. The results showed that these coacervates exhibit dynamic properties, including emergence, decay, building block exchange, and the concentration of functional RNA in its active folded state. Thus, the study presented these fuel-regulated coacervate droplets driven by a reaction cycle as a useful model for investigating the mechanisms of compositional control and functionality in membraneless organelles [83].

**Figure 11:**
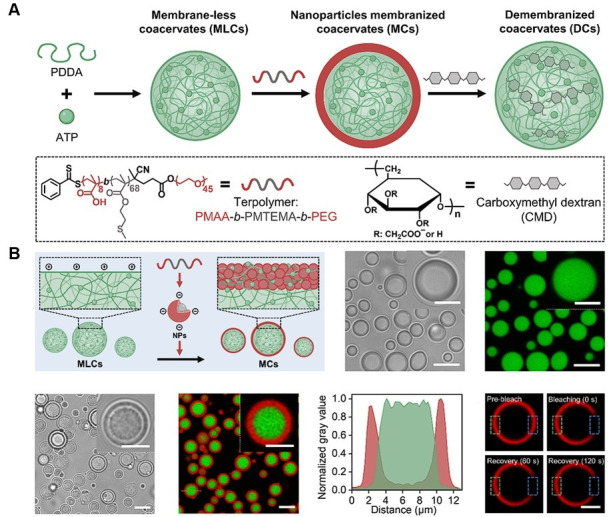
(**A**) Controlled demembranization of membranized coacervate droplets. The membranization of coacervates is achieved by introducing a terpolymer (PMAA-*b*-PMTEMA-*b*-PEG) into MLCs composed of PDDA and ATP. Following this, the demembranization of MCs is triggered by the addition of CMD, which causes the dissociation of the outer membrane and results in DCs integrated with CMD. (**B**) Preparation and characterization of (NR-loaded) NP membranized coacervates. The schematic illustration shows the formation of MCs through the addition of (NR-loaded) NPs. In the presence and absence of NR, the terpolymer initially self-assembles into anionic (NR-loaded) NPs, followed by the membranization on the surface of the cationic MLCs, accompanied by a small partial dissociation of the coacervate phase and the redistribution of the coacervate components into the NP membrane. Bright field and fluorescence field CLSM images of MLCs and MCs, respectively. The scale bar represents 10 μm, and the scale bar in the inserted image represents 5 μm. (Adjusted from ref. [150]. Copyright © 2025 American Chemical Society).

Recent studies have explored not only membraneless protocell models but also strategies to control the membranization and demembranization of protocells, as well as approaches to understanding tissue-level multicompartment systems and biochemical reaction networks through prototissue models. Zhou and colleagues proposed a strategy to regulate the membrane formation (membranization) and membrane removal (demembranization) of coacervate protocells, demonstrating the potential to enhance their functionality and dynamic reconfiguration capabilities [150] (Figure 11). Membraneless coacervates were transformed into membranized structures through coating with terpolymer-based nanoparticles, and the membrane was subsequently removed using the anionic polysaccharide carboxymethyl dextran. This process allowed for structural reconfiguration and controlled permeability toward biomolecules, ultimately leading to the development of synthetic protein-containing protocells with hierarchical and asymmetric membrane architectures.

Meanwhile, Hu and colleagues presented a binary droplet-based protocell network formed from coacervates and aqueous two-phase systems (ATPSs) to mimic prototissues. This network facilitated the spatial self-sorting of biomacromolecules, thereby enhancing biological reactions and material extraction processes. Furthermore, the study highlighted the importance of the dynamic nature of coacervation in organizing intracellular environments and regulating biochemical processes [151].

## 7. Fundamental Challenges of Coacervation

Coacervate systems encounter notable stability issues in biological environments. The main limitation arises from their sensitivity to variations in ionic strength, pH, or temperature [107,199]. This characteristic, while allowing for controlled release properties, creates significant obstacles for systemic delivery routes where the coacervate must traverse various biological environments with different ionic compositions and pH levels. To overcome the inherent limitations while preserving the beneficial properties that make coacervates attractive for drug delivery applications, covalent crosslinking strategies, click chemistry, and multivalent hydrogen bonding have been demonstrated [199,200,201]. The effectiveness of these crosslinking strategies has been validated through dynamic light scattering studies, which demonstrated significant improvements in stability against both salt and pH changes [199]. Post-coacervation crosslinking uses thiolene click chemistry, which enables the formation of covalent crosslinks in aqueous media without exposure to heat or organic solvents [200]. Visual confirmation through optical microscopy has demonstrated that, while both the crosslinked and uncrosslinked coacervates maintain droplet structure at a pH of 6.5, only the crosslinked coacervates preserve their droplet morphology at a pH of 2.0. The enhanced stability achieved through this crosslinking approach extends beyond pH resistance to include improved performance in various biological conditions such as the stomach or inflammatory sites. A dopamine-containing multi-hydrogen bonded peptide complex coacervate exhibiting stability in a pH range of 1–11 and various salt concentration ranges has been developed [201]. These peptide coacervates demonstrated high drug encapsulation efficiency and trypsin-induced release characteristics, showing great potential for oral drug delivery applications.

Recent research has shown that coacervates can function as transient protective structures in vivo or serve as smart platforms for stimulus responsive drug release [202,203]. For example, Zhao et al. developed an oral drug delivery strategy using nanoparticle-assembled bioadhesive coacervates. In a mouse model, the orally administered drug-loaded coacervates significantly alleviated the symptoms of inflammatory bowel disease, restored gut microbiota diversity, reduced systemic drug exposure, and improved therapeutic efficacy in acute colitis models [204]. This system can be administered orally, providing a more patient-friendly alternative to traditional drug delivery methods such as enemas or injections. Additionally, by releasing the drug directly into the gastrointestinal tract in a controlled manner, it minimizes systemic exposure to the drug, thereby reducing the risk of the adverse effects associated with the long-term use of corticosteroids and other drugs that may cause serious side effects, while maintaining therapeutic efficacy. In other studies, Wang et al. and Park et al. utilized coacervation-based hydrogels for wound healing applications and demonstrated enhanced tissue regeneration in mouse models, with wound healing and infection control following treatment. Specifically, the self-healing ability of the hydrogel after being damaged means that it can maintain its functionality over time, reducing the need for frequent dressing changes [163,205]. This can lead to improved patient comfort and reduced healthcare costs associated with the wound management. As a result, coacervation has gained increasing attention in various in vivo applications. The favorable results suggest strong potential for clinical use.

## 8. Future Perspective for Coacervation

Despite encouraging in vitro results, the clinical translation of coacervate-based delivery systems continues to face significant challenges. In particular, their structural instability under physiological conditions remains a major limitation for systemic administration routes such as intravenous or oral delivery [201]. In vivo, coacervates are exposed to various destabilizing factors, including proteolytic enzymes, immune responses, fluctuations in ionic strength, and interactions with the extracellular matrix, all of which can induce phase separation breakdown or premature disassembly. To address these challenges, systematic in vivo studies are essential to evaluate the long-term stability, functional retention, target specificity, and immunogenicity of coacervation [18].

One promising direction is the development of stimuli-responsive coacervates that can remain stable during circulation but disassemble selectively at the target site [206]. For instance, complex coacervate core micelles incorporating reversible crosslinking have shown enhanced control over phase stability and release behavior. Although polyethylenimine is frequently used to generate small, stable coacervates, concerns remain regarding its cytotoxicity and limited transfection efficiency in vivo [203]. In response, low-molecular-weight polyethylenimine grafted with biocompatible polymers such as chitosan has demonstrated improved buffering capacity and cellular uptake with reduced cytotoxic effects, making it a promising alternative [202].

Another critical consideration for clinical trials is the scalability and reproducibility of manufacturing processes. Recent advances in microfluidic-based fabrication, particularly for lipid nanoparticle production in mRNA vaccine platforms, offer a blueprint for adapting similar technologies to coacervate systems. These platforms enable precise control over particle size, composition, and encapsulation efficiency, while maintaining the batch-to-batch consistency required for regulatory approval [9,152].

In addition to engineering advances, gaining mechanistic insight into how coacervates behave in complex biological environments is crucial. Techniques such as confocal laser scanning microscopy, dynamic light scattering, isothermal titration calorimetry, and cryo-electron microscopy are increasingly employed to study phase behavior, structural integrity, and biomolecular interactions under physiologically relevant conditions [207,208,209].

## 9. Conclusions

This review highlights biomolecule-based coacervation as a flexible and dynamic platform that offers a wide range of functions in the biomedical and biotechnological fields, including drug encapsulation, self-assembly, and the development of functional materials. Biomolecule-based coacervates provide a favorable environment for handling sensitive biological molecules such as proteins, RNA, and enzymes, owing to their inherent biocompatibility, tunable composition, and mild assembly conditions. Recent studies have demonstrated the applicability of these systems in diverse areas such as the controlled release of therapeutics, gene editing platforms (e.g., Cas9, siRNA, and mRNA), wound healing scaffolds, and protocell-based synthetic biological architectures. However, several challenges remain before these coacervate systems can be fully translated into clinical or industrial settings. Major hurdles include mechanical instability under physiological conditions, insufficient droplet uniformity and reproducibility, and a limited understanding of long-term biodegradability and biocompatibility. The lack of a well-established regulatory framework for therapeutic applications is also a significant barrier. To overcome these challenges, integration with advanced technologies such as microfluidics, AI-assisted material design, and 3D bioprinting is required to enable precise spatial control, large-scale production, and clinical translation. In parallel, convergence with systems biology, omics-based analysis, and high-throughput screening platforms will be essential to elucidate the mechanisms of interaction between the coacervates and cells, and to develop application-specific design strategies. With continued technological refinement and interdisciplinary collaboration, biomolecule-based coacervation is expected to play a central role in a variety of fields, including precision medicine, synthetic cell engineering, next-generation therapeutics, functional food and vaccine platforms, and the construction of spatially controlled biological environments.

## Data Availability

Dataset available on request from the authors.

## Abbreviations

The following abbreviations are used in this manuscript:

ApoEV	Apoptotic extracellular vesicle
ATP	Adenosine 5′-triphosphate
BMP-2	Bone morphogenetic protein 2
BMSC	Bone marrow mesenchymal stem cell
CLSM	Confocal laser scanning microscope
CMC	Carboxymethyl cellulose
CMCS	Carboxymethyl chitosan
CMD	Carboxymethyl dextran
CS	Chitosan
DC	Demembranized coacervate
ECM	Extracellular matrix
ELP	Elastin-like polypeptide
FG	Fish gelatin
GA	Arabic gum, gum arabic
GAG	Glycosaminoglycan
HA	Hyaluronic acid
HMWC	High-molecular-weight chitosan
HMWG	High-molecular-weight cationized gelatin
HRV	Rhinovirus
IGF-1	Insulin-like growth factor-1
LLPS	Liquid–liquid phase separation
LMWC	Low-molecular-weight chitosan
LMWG	Low-molecular-weight cationized gelatin
MAP	Mussel adhesive protein
MC	Nanoparticle membranized coacervate
MLC	Membraneless coacervate
OVA	Ovalbumin
PDDA	Poly(diallyldimethylammonium chloride)
PLL	Poly-L-lysine
PP	Pea protein
PPV	Procine parvovirus
QHECE	Hydroxyethylcellulose ethoxylate
RNP	Ribonucleoprotein
ROS	Reactive oxygen species
SA	Sodium alginate
SPI	Soy protein isolate
SPP	Soluble pea protein
SSPS	Soluble soybean polysaccharide
WPI	Whey protein isolate
